# Blending Proteins in High Moisture Extrusion to Design Meat Analogues: Rheological Properties, Morphology Development and Product Properties

**DOI:** 10.3390/foods10071509

**Published:** 2021-06-30

**Authors:** Patrick Wittek, Heike P. Karbstein, M. Azad Emin

**Affiliations:** Institute of Process Engineering in Life Sciences, Food Process Engineering, Karlsruhe Institute of Technology, 76131 Karlsruhe, Germany; patrick.wittek@kit.edu (P.W.); heike.karbstein@kit.edu (H.P.K.)

**Keywords:** high moisture extrusion, meat analogue, soy protein, whey protein, protein blends, rheological properties, morphology development, texturisation

## Abstract

High moisture extrusion (HME) of meat analogues is often performed with raw materials containing multiple components, e.g., blends of different protein-rich raw materials. For instance, blends of soy protein isolate (SPI) and another component, such as wheat gluten, are used particularly frequently. The positive effect of blending on product texture is well known but not yet well understood. Therefore, this work targets investigating the influence of blending in HME at a mechanistic level. For this, SPI and a model protein, whey protein concentrate (WPC), were blended at three different ratios (100:0, 85:15, 70:30) and extruded at typical HME conditions (55% water content, 115/125/133 °C material temperature). Process conditions, rheological properties, morphology development, product structure and product texture were analysed. With increasing WPC percentage, the anisotropic structures became more pronounced and the anisotropy index (*AI*) higher. The achieved *AI* from the extrudates with a ratio of 70:30 (SPI:WPC) were considerably higher than comparable extrudates reported in other studies. In all extrudates, a multiphase system was visible whose morphology had changed due to the WPC addition. The WPC led to the formation of a much smaller dispersed phase compared to the overlying multiphase structure, the size of which depends on the thermomechanical stresses. These findings demonstrate that targeted mixing of protein-rich raw materials could be a promising method to tailor the texture of extruded meat analogues.

## 1. Introduction

High moisture extrusion (HME) can be used to produce meat analogues from plant-based, protein-rich raw materials that are intended to mimic meat in terms of its unique structure, texture, and mouthfeel [1,2]. The studies on HME, which started in the 1980s [3,4,5], can basically be classified into two different categories, which refer to the use of the raw material, either single-component, where only a single protein-rich raw material (e.g., a protein concentrate or isolate) was used [3,6,7,8,9,10,11,12], or multi-component, where two or more raw materials were mixed together [13,14,15,16,17,18,19,20,21]. For both categories, it was shown that anisotropic meat-like structures were created in the process. Nevertheless, the multi-component systems tended to have a more meat-like product structure and texture compared to the single-component systems [1,18]. Different factors were suggested to be the cause of this effect, such as the formation of a dispersed phase from the added component [1,22,23], the influence of the component on protein aggregation/crosslinking reactions [17,18], or changes of the rheological properties [19]. However, there is still a lack of understanding of what the positive effect of adding a second protein-rich raw material in HME on the product structure is based on. 

In our previous work, we showed that a multiphase system is formed in the single-component extrudates based on soy protein isolate (SPI) in which the phases differ in their water concentration [10]. According to the model of Tolstoguzov [24], this multiphase system arises as a result of the thermodynamic incompatibility of the proteins involved. The compatibility between proteins is determined by their differences in hydrophilicity, the ratio of molecular weights, and their conformational state [25,26]. This phase separation can also occur in a single component system such as SPI [10], which for instance naturally involves more than two protein fractions (e.g., ß-conglycinin and glycinin) [27] with different hydrophilicity. These differences in hydrophilicity lead to the apparent phase-dependent water concentration, where the phase with the more hydrophilic proteins has a higher water concentration [26].

Due to the thermomechanical stresses in the extrusion process, the morphology of the multiphase system is subjected to changes, as, it can deform, break up and/or coalesce [28]. The rheological properties therefore play a major role, which in turn depend on the composition of the proteins respectively the raw material [29]. For example, Dekkers et al. [30] were able to show in a highly concentrated system that the addition of wheat gluten led to a reduction in the storage modulus G′ of SPI.

If these findings on phase separation and phase behaviour in highly concentrated protein systems are taken as a basis, it can be expected that the admixture of another protein-rich raw material will affect the morphology, water distribution and rheological properties of the system and therefore lead to a change in the product structure and texture.

In order to better understand these interrelationships, we blended two protein-rich raw materials in this study. The main raw material is soy protein isolate, which is known to form anisotropic structures as a single component in HME [10,12,31]. Whey protein concentrate (WPC) is used as the protein-rich model raw material to be added to the SPI and is admixed in two concentrations (15 and 30%) to obtain three mixing ratios: 100:0, 85:15 and 70:30. Whey proteins were chosen as a model protein because, compared to soy proteins, they possess a distinctively different amino acid composition [32], which should enhance the differences in hydrophilicity and therefore the effect on the system. Additionally, a mixture of plant and animal proteins could prove advantageous for the product properties. Blending can increase nutritive value by complementing amino acid profiles [33,34], as plant-based proteins often have fewer indispensable amino acids and therefore lower PDCAAS-scores [35,36]. It can also improve sensory and textural properties, as proteins from animal origin generally have less off-flavor [37] and specifically, whey proteins were shown to improve creaminess and mouthfeel by acting as a fat replacer [38].

The influence of the added whey proteins on the rheological properties of the blends was investigated under extrusion-like conditions. The blends were extruded at different temperatures and the influence of the whey proteins on the process conditions, the anisotropic product structure, the extrudates morphology development respectively water distribution and the textural properties were determined. In addition, the phase behaviour and morphology of the whey proteins in the overall matrix were investigated.

## 2. Material and Methods

### 2.1. Material

Commercial soy protein isolate Supro ST from Solae LLC (St. Louis, MO, USA) was used for this work. The manufacturer specifies a protein content of at least 90% on a dry basis. The contents of the minor ingredients are ≤1% fat and ≤5% ash. The moisture content of 3.4% (*w*/*w*) was determined gravimetrically. Commercial whey protein concentrate GermanProt 8000 was kindly provided by Sachsendorf Leppersdorf GmbH (Leppersdorf, Germany). According to the manufacturer, it has a protein content of at least 80% on a dry matter basis. The contents of the minor ingredients are ≤8.5% lactose, ≤6.5% fat and ≤4% ash. The gravimetrically determined moisture content was 5.4% (*w*/*w*).

### 2.2. Extrusion Trials

Extrusion trials were conducted using a co-rotating twin-screw with a screw diameter of 11 mm (Process 11, Thermo Fisher Scientific, Waltham, MA, USA). The length to diameter (L/D) ratio is 40 and a slit cooling die was attached to the extruder through a die adapter. Only forward elements were used in the screw configuration.

The extruder barrel consists of eight barrel elements. Besides the first barrel, all barrels can be heated and cooled independently. The die adapter can also be heated. Solids are dosed via a gravimetrically controlled feeder from Brabender Technology GmbH (Duisburg, Germany) in the first barrel element, and water is dosed via a peristaltic pump from Cole Parmer (Masterflex L/S, Vernon Hills, IL, USA) in the third barrel element. The die adapter is 32 mm long and provides a transition from the screw section to the cooling die. The cooling die (125 × 19 × 4 mm) was cooled with a temperature control liquid Thermal HL60 at −10 °C, which was supplied by a water-cooled process circulator Presto Plus LH 47, both from Julabo GmbH (Seelbach, Germany).

The screw speed was kept constant at 600 rpm during the extrusion trials. A 0.9 kg/h protein blend and 1.1 kg/h water (tap water with a conductivity of 584.75 ± 16.78 µS/cm from Karlsruhe, Germany) were always added. The SPI + WPC blends were premixed outside the extruder; for a WPC concentration of 15%, 0.135 kg/h WPC and 0.765 kg/h SPI were added, for a WPC concentration of 30%, 0.27 kg/h WPC and 0.63 kg/h SPI were added. Taking into account the raw material moisture, this results in moisture contents of 56.5% (SPI), 56.7% (SPI + 15% WPC) and 56.8% (SPI + 30% WPC) (*w*/*w*) for the protein blends in the extrusion trials.

The temperature setting for the barrel elements 2 to 5 was: T_Barrel,2_ = 25 °C, T_Barrel,3_ = 50 °C, T_Barrel,4_ = 90 °C, T_Barrel,5_ = 110 °C. The barrel elements 6 to 8 and the die adapter were specifically adjusted to achieve one of the three desired material temperatures (115, 125, 133 °C) for each material system. The temperature profiles required for this are given in Table 1.

The material temperature and the die pressure were measured in the die adapter mounted prior to the cooling die. Sampling for further analysis was performed once the die pressure and material temperature were constant for at least three minutes. The pressure was then measured for three more minutes and averaged. To investigate the anisotropic product structure, the extrudates were torn open and photographed immediately after exiting the extruder. For further analysis, the extrudates were immediately vacuumed after exit, frozen and stored at −18 °C until further use.

### 2.3. Texture Analysis

The textural properties of the extrudates were determined via cutting measurements, which are derived from previous work on HME [12,39]. The measurements were performed with a materials testing machine from Zwick&Roell (zwickiLine Z2.5, Ulm, Germany). Before measurement, the extrudates were thawed at room temperature. Then rectangular pieces were punched out with a punch geometry (8.5 × 15 mm), longitudinally and transversely to the flow direction. The punched pieces were then cut 3.8 mm deep (95% of the extrudate thickness) along the long side with an in-house manufactured knife blade (A/LKB), at a cutting speed of 0.5 mm/s. The cutting force was recorded and the maximum cutting force was determined and selected as representative of the sample. Each cut per cutting direction and extrudate was repeated at least 12 times. The anisotropy index (*AI*) was calculated from the ratio of the transversal cutting force (F_T_) to the longitudinal cutting force (F_L_): *AI* = F_T_/F_L_.

### 2.4. Cryo-Imaging

The morphology of the extrudates was analysed by cryo-imaging [10]. For this purpose, the frozen samples were cut laterally to the flow direction (side view) with a cryo-microtome (CM 3050, Leica Biosystems GmbH, Nussloch, Germany). A cooling room temperature of −14 °C and a sample holder temperature of −12 °C were set for the instrument. To prepare the extrudates for cutting, 4–5 cm long sections were cut out from the extrudate strand. These sections were attached to the sample holder with a sectioning medium from Leica Biosystems GmbH (FSC 22 Frozen Section Media, Nussloch, Germany). This ensured product stability on the sample holder during sectioning and had no influence on the product microstructure. By cutting 40-µm sections from the extrudate, smooth cut surfaces were obtained. Photos of the cut surface were taken immediately after cutting of the extrudate in the cryo-microtome with a digital camera (DMC-GH2, Lumix, Kadoma, Japan) and a 4608 × 3456 pixel resolution.

### 2.5. Micro-CT

The morphology of the extrudates was also analysed by measurements with a micro-computed tomograph (micro-CT) [10]. The preparation of samples for the X-ray measurements in the micro-CT was initiated by freeze-drying 5–6 cm sections of the extrudate strand with a lab-scale freeze-dryer from Martin Christ Gefriertrocknungsanlagen GmbH (Alpha 1–4 LDplus, Osterode, Germany) at −80 °C and 50 mbar for at least 72 h. The dried samples were then analysed with the Xradia 520 from Carl Zeiss Microscopy Deutschland GmbH (Oberkochen, Germany). The parameters of the measurements were: 0.4× objective, 2× binning, source filter: air, 50 kV voltage, 4 W power. The exposure time was adapted for each extrudate and scanning position, depending on the measured transmittance, to ensure image quality, and varied between 0.8 and 2.4 s. A full 360°-scan with 801 projections was performed.

### 2.6. Rheological Measurements

**Figure 1:**
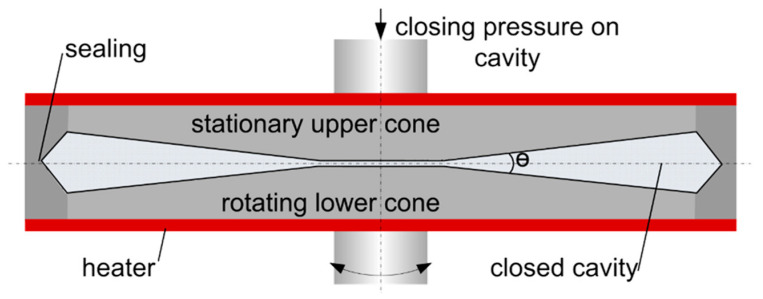
Closed cavity rheometer used for rheological characterisation of the material (picture taken from Emin and Schuchmann, 2017 [42]).

To determine the rheological properties of the proteins blends at extrusion-like conditions, samples were analysed in a closed cavity rheometer (CCR) from TA Instruments, Inc. (RPA flex, New Castle, DE, USA); see Figure 1. A detailed description of this instrument can be found elsewhere [40,41]; only a brief description should be given here. The sample (5.5–6.0 g) is initially placed between the two cones. Then, the upper cone moves downwards and applies a closing pressure. This creates a closed and pressurised cavity for the material and therefore prevents water evaporation at high temperatures. The rheological properties can then be calculated from the resulting force of the sinusoidal rotary deformation of the lower cone.

For the dough preparation, solid SPI and WPC were first mixed at ratios of 100:0, 85:15 and 70:30, which resembles the 0, 15 and 30% of WPC in the material systems. The solid blends were then mixed with deionised water in a Thermomix from Vorwerk (Wuppertal, Germany) to achieve a moisture content of 55% *(w/w)* for all mixtures, taking into account the raw material moisture. The doughs were then vacuum sealed and stored in a refrigerator at 4 °C for at least 12 h prior to the measurements to ensure uniform water distribution and hydration.

The measurement routines were performed at three temperatures: 115, 125 and 133 °C. In each routine, the material was treated for 60 s at the desired temperature and a frequency of 1.0 Hz and 0.98% to ensure the constant temperature of the sample. The deformation parameters are well in the linear viscoelastic range (LVE) of the materials, where rheological properties are not stress or strain-dependent [43], and therefore no influence of the measurements mechanical treatment on the material is expected. Afterward, a strain sweep at the same desired temperature and a constant frequency of 1.0 Hz was performed. The strain increased gradually from γ = 0.9–1005%. Storage modulus G′ and loss modulus G″ were determined as a function of strain γ.

### 2.7. Scanning Electron Microscopy (SEM)

The analysis of the phase behaviour and morphology of the whey proteins was performed with SEM measurements. For this purpose, materials were first treated in the CCR and afterward examined with the SEM. Doughs of SPI and SPI + 30% WPC with a moisture content of 55% *(w*/*w)* were prepared as described for the rheological measurements. 

For the determination of the influence of thermomechanical treatment, samples were treated at 125 °C for 60 s and an additional oscillatory deformation. For SPI, only an oscillatory deformation in the LVE at 1.0 Hz and 0.98% was performed, which equals and will be described as “0 s^−1^”. For SPI + 30% WPC, three different oscillatory deformations were applied: 1.0 Hz and 0.98% (0 s^−1^), 10.0 Hz and 120% (which equals 75 s^−1^) and 10.0 Hz and 240% (150 s^−1^). After the one-minute treatment, the material was cooled to 90 °C in the CCR, the cavity opened and the pellet was taken out.

For the determination of the influence of singular rotatory shear deformation, samples from SPI + 30% WPC were treated at 125 °C for 60 s and an oscillatory deformation in the LVE at 1.0 Hz and 0.98%. Afterward, the material was cooled to 90 °C. At 90 °C, the material was deformed in a continuously rotatory way, with a constant strain of 5000% (which is almost a 360° rotation) but at different deformation times. A decrease in time means an increase in the shear rate. Deformation times of 200 s (0.25 s^−1^), 50 s (1 s^−1^), 20 s (2.5 s^−1^) and 5 s (10 s^−1^) were applied. After this deformation, the cavity was opened and the pellet was retained. The formation of this pellet from raw material dough is shown in a recent study [38].

**Figure 2:**
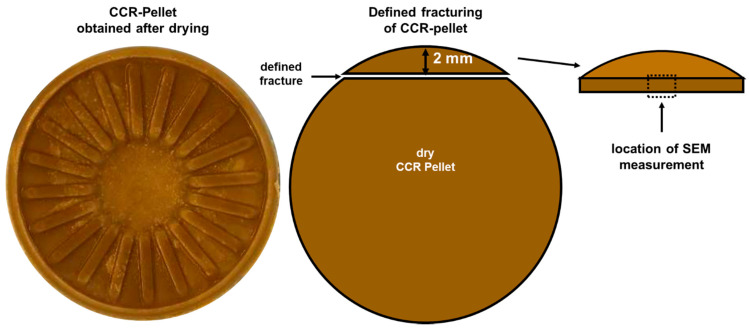
Sketch of the process of preparing the CCR-pellets for SEM measurements.

The pellets from both routines were prepared for the SEM measurements by pre-drying for at least 72 h at 25 °C in room air, and subsequently by vacuum-drying for at least 72 h at 25 °C and 10 mbar. To reveal the inner structure of the pellet, these pellets were broken at a defined distance of 2 ± 0.1 mm from the outside radius, as shown in Figure 2.

To prepare the fractured surface for SEM measurement, a high vacuum sputter coater from Leica Microsystems GmbH (EM ACE 600, Wetzlar, Germany) applied a 7 nm platinum coating and the coating was contacted with conductive silver. The SEM measurements were performed with an environmental scanning electron microscope from FEI (Quanta 650 FEG, Eindhoven, The Netherlands), using secondary electrons, topographical contrast and an Everhart-Thornley detector.

## 3. Results and Discussion

### 3.1. Influence of Protein Blending on Process Conditions

**Figure 3:**
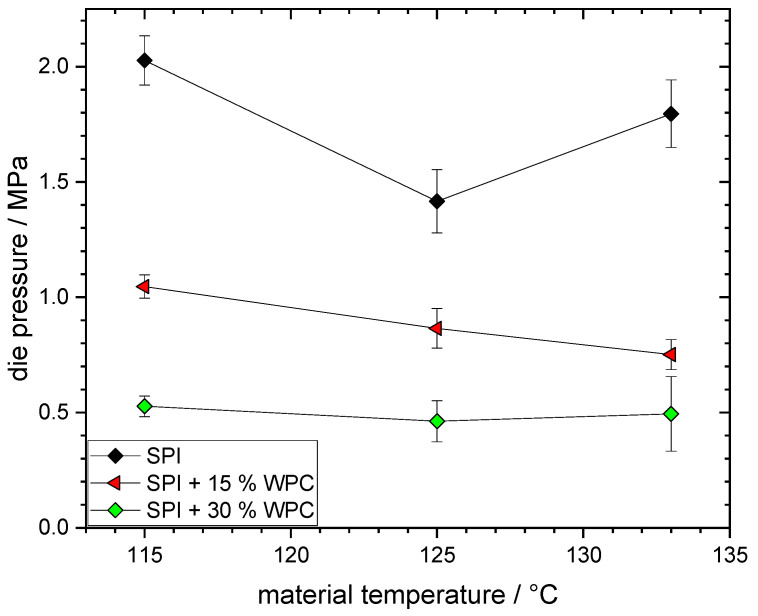
Die pressure as a function of material temperature for different protein blends (0–30% WPC).

The die pressure values for the different protein blends are plotted above the material temperature at the extruder die in Figure 3. The lines between the points were drawn to guide the eye. Total mass flow and die geometry were kept constant in these trials.

The addition of WPC has a distinct effect on the die pressure: an increase in WPC concentration results in a decrease in die pressure. For SPI, the values range from 1.4 MPa at 125 °C to 2.0 MPa at 115 °C. With 15% WPC, the die pressure reaches a range of 0.8 to 1.0 MPa. At 30% WPC, the die pressure is constant at about 0.5 MPa.

As the total mass flow and the die geometry are kept constant, the die pressure is a direct function of the viscosity of the material. Thus, the reduction in pressure due to the addition of WPC indicates a reduction in viscosity. This could be due to two effects: firstly, a mixing effect when the rheological properties of the mixture are a simple combination of those of the individual components WPC and SPI. Secondly, a dispersed phase could have been formed, which influences the rheological properties through a “weakening” of the overall structure (“dispersed phase effect”). This effect of a dispersed phase is known, for example, in polymer blends [44], where even small amounts of a second polymer change the rheological properties beyond the mixing effect.

The reduction in pressure with increasing temperature, as occurs at SPI between 115 °C and 125 °C and at SPI + 15% WPC for all three temperatures, can be explained by a reduction in viscosity due to increased mobility of the molecules [45]. At least a small reduction in die pressure would have been expected at SPI + 30% WPC; thus, this expected viscosity reduction must have been outweighed by some other effect. Whey proteins are known to be reactive and aggregate at extrusion conditions [46,47], leading to an increase in viscosity [48], which could be such an overlying effect. Reactivity could also be considered for the pressure increase at 133 °C in SPI. However, Wittek et al. [10] have shown that the present SPI is not reactive at comparable extrusion conditions. Thus, the reason for the pressure increase with SPI remains unclear.

### 3.2. Influence of Protein Blending on Product Structure and Texture

**Figure 4:**
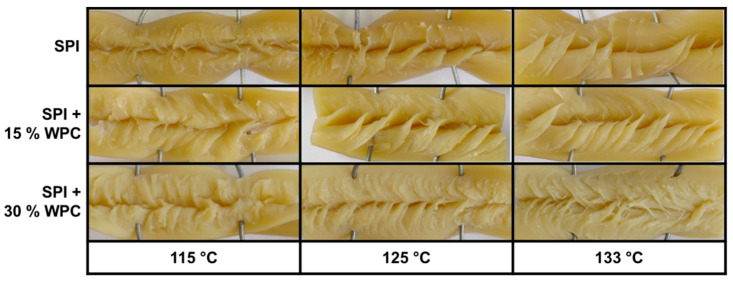
Product structure of extrudates for different protein blends (0–30% WPC) and material temperatures (115–133 °C).

The product structure of the extrudates for the different material temperatures and WPC concentrations can be seen in Figure 4.

An anisotropic structure was formed for all protein blends at all investigated process conditions. The ability of sole SPI to form anisotropic structures in HME has already been demonstrated under comparable conditions [10,12], as well as for blends of SPI and WPC [49]. 

The addition of 15% WPC leads to a more pronounced anisotropic product structure for all three material temperatures. The effect of blending protein-rich raw materials on the product structure and/or anisotropy has already been reported [1,18].

Further increase in the WPC concentration to 30% also has an effect on the anisotropic structure. In this case, the anisotropic structures are more pronounced than with SPI, and the structures are thinner than in SPI + 15% WPC. Park et al. [15] demonstrated that increasing wheat gluten content improved the textural properties of extruded SPI, but it is not reported how the underlying anisotropic structures developed.

An increase in the material temperature leads to a more pronounced anisotropic structure in all three protein blends. In a study with the same SPI and comparable process conditions [10], it was suggested that this was due to the dependence of the rheological properties on temperature [45].

**Figure 5:**
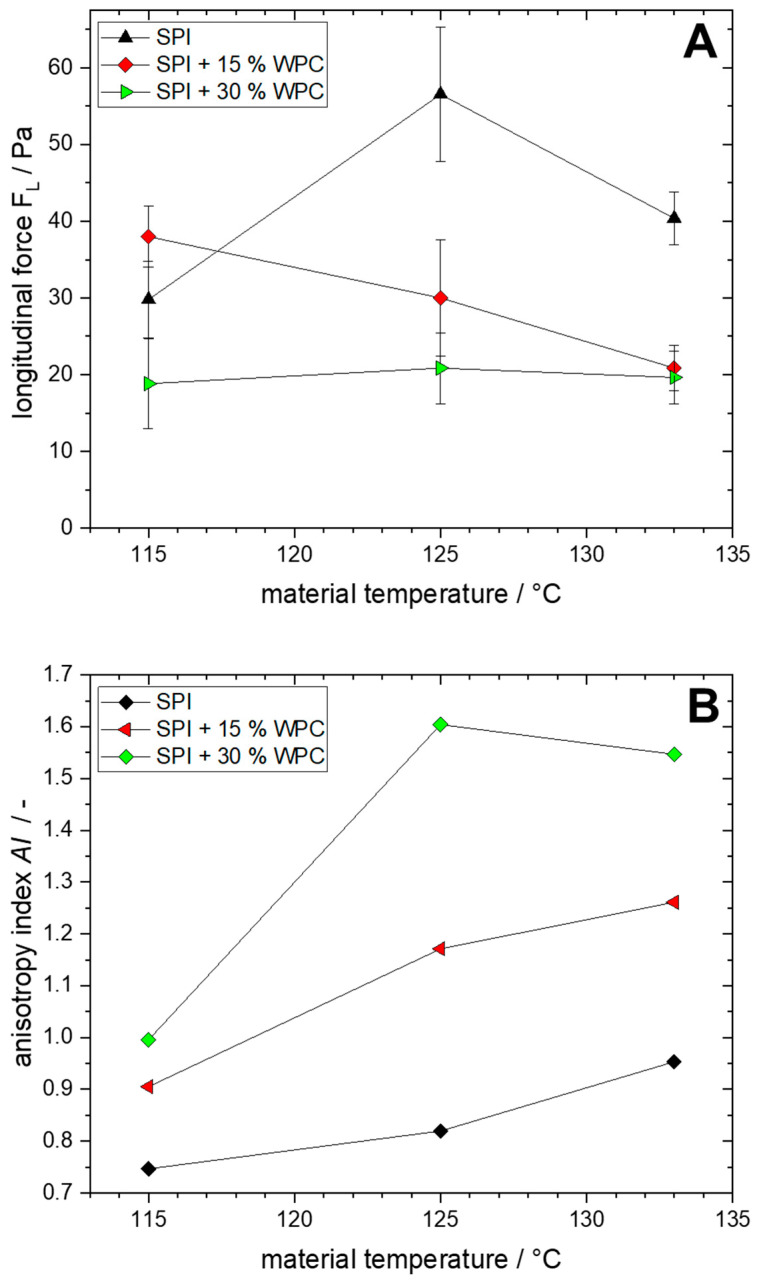
Longitudinal force (**A**) and anisotropy index *AI* (**B**) as a function of material temperature for different protein blends (0–30% WPC).

The textural properties of the extrudates, i.e., longitudinal force and anisotropy index (*AI*), can be seen in Figure 5. The lines between the points were drawn to guide the eye.

For both parameters, the influence of WPC addition can be clearly seen. For the longitudinal cutting force, no clear correlation to the WPC concentration is apparent at 115 °C, it ranges from 19 to 38 Pa. However, at 125 °C, 15% WPC leads to a reduction of the cutting force from 57 to 30 Pa, while at 30% WPC, it is only 21 Pa. For 133 °C, the cutting force also decreases due to WPC addition, from 40 Pa to 21 Pa with SPI + 15% WPC and 20 Pa with SPI + 30% WPC.

With increasing temperature, no distinct trend can be seen: for SPI, the cutting force first increases and then decreases. For SPI + 15% WPC, the cutting force decreases with increasing temperature, while for SPI + 30% WPC it remains almost constant.

For the anisotropy index (*AI*), as for the longitudinal cutting force, the large influence of the WPC addition is apparent. As the WPC concentration increases, the *AI* increases, with a smaller relative increase at 115 °C (from 0.75 to 0.91 to 1.0) than at 125 °C (0.82 to 1.17 to 1.60) and at 133 °C (0.95 to 1.26 to 1.54). Likewise, the *AI* increases with increasing temperature, except for the step from 125 °C to 133 °C at SPI + 30% WPC, where it decreases slightly.

Different studies on HME have also used cutting tests to characterize the textural properties, but a comparison of the absolute values for the cutting values is hardly possible, since the measurement parameters, such as blade sharpness, were not the same. However, the *AI* can be compared, since here the measurement setup-specific influences are eliminated.

For the extrusion of three different pea protein isolates, it could be shown as well that the *AI* is influenced by the material temperature [39]. For extruded soy protein concentrate, the *AI* was found to be 1.04 [50], and for blends of peanut protein, SPI, and wheat gluten in various ratios, the *AI* ranged from 0.91 to 1.06 [17]. For single-component SPI, *AI* ranged from circa 0.9 to 1.2 at different process conditions [12]. Thus, these values are comparable to those obtained in this work for SPI and SPI + 15% WPC, but considerably higher *AI* (1.54 and 1.60) are obtained for SPI + 30% WPC.

The texture measurements as a method to quantitatively describe the anisotropy of the products also correlates with the visual appearance of the anisotropic structures from Figure 4. With increasing WPC concentration and temperature, the structure becomes more pronounced and thinner, which is also reflected in the increase in *AI*.

### 3.3. Morphology Development in Extrudates

**Figure 6:**
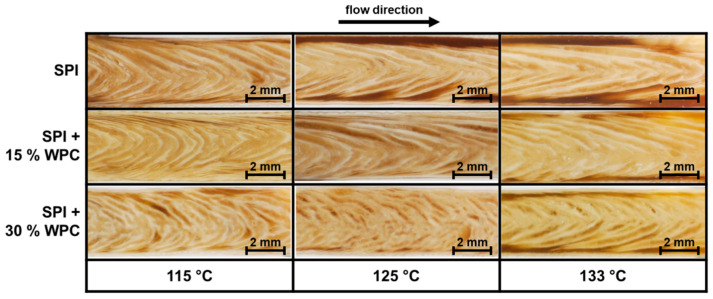
Multi-phase morphology, obtained through cryo-imaging of extrudates for different protein blends (0–30% WPC) and material temperatures (115–133 °C).

The morphology development in the extrudates, visualised by cryo-imaging, can be seen in Figure 6. The flow direction is from left to right.

All extrudates show a clearly visible multiphase system, with the phases differing in color and brightness. The phases are always deformed in the direction of flow, as already shown by Wittek et al. [10], and have the shape of an assumed flow profile. In the following, this shape will be called the “V-shape”. The individual enclosed structures themselves exhibit a high degree of anisotropy, i.e., they are significantly longer than they are wide (L/D > 5).

The addition of 15% WPC has only a minor effect on the morphology, with the structures becoming slightly thinner. When increasing to 30% WPC, the influence is more pronounced. The structures are more disordered, shorter and thinner than with the other two WPC concentrations. In addition, as the WPC concentration increases, the angle of the V-shape decreases.

Temperature also has a significant effect on morphology. For all three WPC concentrations, the angle of the V-shape increases with increasing temperature.

**Figure 7:**
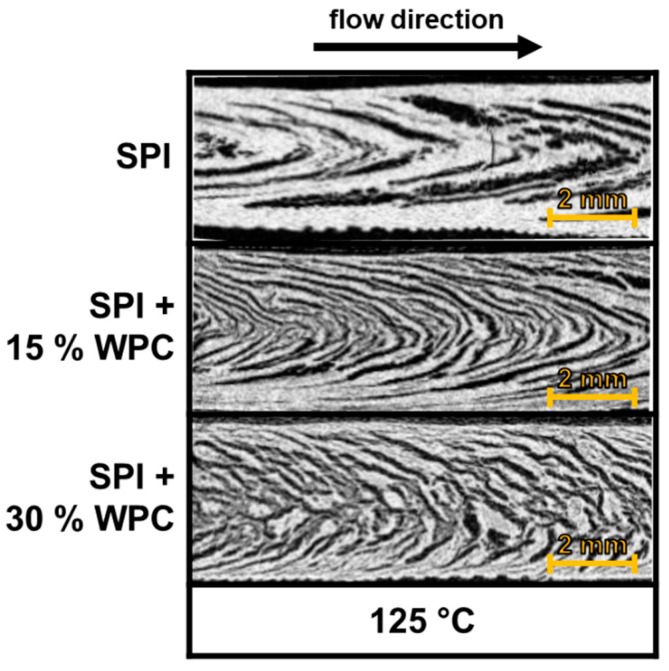
Multi-phase morphology, obtained through micro-CT of freeze-dried extrudates, for different protein blends (0–30% WPC) and material temperature of 125 °C.

The multiphase morphology of the freeze-dried extrudates for a material temperature of 125 °C, visualised by micro-CT, can be seen in Figure 7. The flow direction is from left to right.

The dark areas indicate low density, while the light areas indicate high density, relatively speaking. The morphology shown here is almost identical to that from cryo-imaging (Figure 6). The characteristic V-shape can be seen for all three WPC concentrations and the angle of the V-shape decreases with increasing WPC concentration. In addition, as seen in the cryo-imaging images, the (dark) dispersed phase be.

The multiphase system arises as a result of the thermodynamic incompatibility of the proteins [24]. As discussed in earlier work from us [10], the results indicate that a water-rich dispersed phase is present and distributed in a water-poor continuous phase. The distribution of water can be attributed to the different hydrophilicities of the proteins involved [26]. Since proteins of different origins differ in their amino acid composition [32], it is also expected that the added whey protein, or its fractions (ß-lactoglobulin, α-lactalbumin, etc.), will have different hydrophilicity than the present soy protein fractions. Consequently, the WPC addition must also have a direct influence on the water distribution in the system and so on the water concentration in the individual phases.

Thus, an influence on the morphology development would also be given, since this depends, e.g., via the viscosity ratio, on the rheological properties of the phases involved, which in turn are a function of the particular phase water concentration.

The changes of the rheological properties of the individual phases through the water distribution are a very likely explanation for the differences in the morphology development, as they would strongly affect the deformation forces acting on the phases. However, another effect might have played an additional role; the decreasing die pressure values with increasing WPC concentration suggest that the added WPC also causes a direct influence on the rheological properties of the overall system, as already discussed, for example, via the mixing effect or the dispersed phase effect. This might also influence morphology development.

### 3.4. Influence of Protein Blending on Rheological Properties

**Figure 8:**
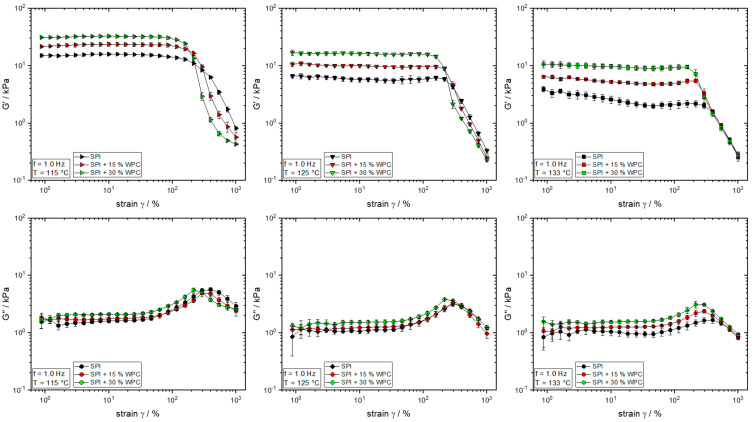
Storage modulus G′ and loss modulus G″ as a function of strain γ for different protein blends (0–30% WPC) and temperatures (115–133 °C).

The rheological properties of the protein blends with different WPC concentrations were characterised by means of strain sweeps at the different material temperatures of the extrusion trials. The results can be seen in Figure 8, where G′ and G″ are plotted above the strain, respectively.

For almost all investigated protein blends and temperatures, a plateau of G′ is visible for low strains. From a certain point, which marks the end of the LVE, G′ drops sharply; this marks the beginning of the non-linear viscoelastic range (nLVE). The rheological behaviour in the nLVE was suggested to be relevant for the behaviour in the extrusion process, where high shear rates (and thus high strains) occur [41,51].

For the curve of G″, all materials show a so-called “weak strain overshoot”: that is, an increase in G″, reaching a maximum, and then the decrease in G″. This behaviour was shown for different highly concentrated biopolymers [41,51,52] and may have different causes; however, a clear structural interpretation is difficult [53] and will not be further discussed here.

In the LVE of G′, i.e., for low strains, G′ and G″ increase with increasing WPC concentration. This behaviour is apparent for all temperatures. The range of LVE decreases with increasing WPC concentration. In the nLVE, i.e., for high strains, the trends reverse for 115 and 125 °C, i.e., the G′ decreases with increasing WPC concentration. For 133 °C, the curves then line up. Similar trends as for G′ can also be observed for G″ in the nLVE.

These differences in the behaviour of a material in the LVE and nLVE have already been observed in multiphase polymer blends [54]. Generally, the rheological properties of a blend are related to the morphology of the dispersed phase(s). When the strain of a measurement affects the morphology (which occurs at sufficiently high strain), the rheological properties also change. This can be demonstrated with an example: in a polymer blend of EPDM rubber (ethylene propylene diene monomer rubber) with poly(vinylidene-*co*-hexafluoropropylene), the addition of the second polymer resulted in a significant reduction in shear viscosity in a steady-shear flow field (capillary rheometer) [44], but similar values of complex viscosity were achieved in the LVE through dynamic-oscillatory measurements [55]. The results thus suggest that the WPC has formed a dispersed phase in the extrudate, which triggers the observed rheological behaviour.

The influence of the high strains during the measurements in the nLVE on the phase morphology explains why the increase in G′ and G″ in the LVE through WPC-addition is reversed in the nLVE. Since the rheological behaviour in the nLVE is more representative of the material behaviour in the extruder [51], the lower die pressure, ergo the lower viscosity, can also be explained by the addition of WPC. For the significantly higher shear rates in the extrusion process and in the screw section, respectively, it is expected that the G′/G″-reducing effect due to the addition of WPC is more pronounced.

It is expected that the influence of the WPC addition on the rheological properties of the overall system also has an effect on the morphology development, because, as already mentioned, it strongly depends on the rheological properties [56]. However, only a general statement can be made here. Although the rheological properties of the overall system (continuous and dispersed phase together) can be determined with the methodology used, the viscosity ratios of the phases in the extrudates remain unknown.

### 3.5. Phase Behaviour and Morphology of Whey Protein

The rheological measurements suggest that the WPC has formed a dispersed phase. In the images from the cryo-imaging, it can be seen that the WPC has an effect on the morphology, but not whether and how it is present as a dispersed phase. However, the morphology has not changed fundamentally, i.e., no “new” dispersed phase on the size of the apparent dispersed water-rich phase was formed. Therefore, it was concluded that if the WPC did form a dispersed phase, it would have to be significantly smaller than the over-lying water-rich dispersed phase and thus not visualisable by cryo-imaging.

**Figure 9:**
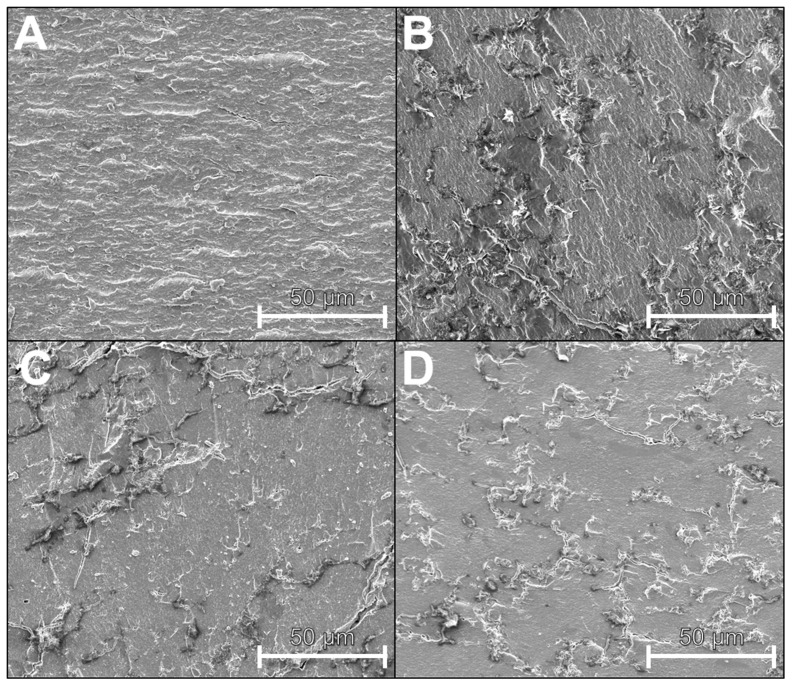
SEM-images of pellets, which underwent defined, extrusion-like thermomechanical treatment at 125 °C and different shear rates (0, 75, 150 s^−1^) in the CCR. (**A**) SPI, 0 s^−1^; (**B**) SPI + 30% WPC, 0 s^−1^; (**C**) SPI + 30% WPC, 75 s^−1^; (**D**) SPI + 30% WPC, 150 s^−1^.

Therefore, SPI and the blend of SPI + 30% WPC were subjected to defined thermomechanical stress in a CCR at extrusion-like conditions (temperature, water content), and the resulting pellet was examined by scanning electron microscopy (SEM). In the first step, the mixture was only to be thermally treated in order to obtain the largest possible dispersed WPC phase, which would facilitate visualisation and serve as a starting and comparison point. Only in the second step, mechanical stress was applied in order to be able to investigate its influence in comparison with the sample treated only thermally. The results of SEM measurements are shown in Figure 9.

In A, the pellet only consists of SPI. An irregular surface in a light grey tone can be seen. The roughness is due to the fracturing of the pellets. After the addition of 30% WPC, dark spots or shapes in the order of 1–20 µm can be seen, distributed in a light matrix (B). It can be assumed that the lighter constituent is the SPI and the dark/black shapes are formed by the WPC, thus showing that a dispersed WPC phase was created.

In C and D the effect of additional mechanical stress on the mixture is shown. The size of the dispersed WPC phase decreases with increasing shear rate and reaches a size of <10 µm for 150 s^−1^. It can therefore be assumed that this dark, dispersed WPC phase is broken up by the mechanical stresses.

This sensitivity of the WPC phase on mechanical stresses also has implications for the extrusion process. In the screw section, even higher shear rates occur than in the CCR. Shear rates of up to 5000 s^−1^ are reported [57,58]. Thus, it can be expected that the dispersed WPC phase will be even more broken up and sizes of <1 µm can be reached. As a result, the dispersed WPC phase would be much smaller than the over-lying dispersed water-rich phase that dominates the morphology in the cryo-imaging images (Figure 6).

In order to better understand the morphology development of the dispersed WPC phase, the system of SPI + 30% WPC was cooled down to 90 °C after a defined thermal treatment at 125 °C and a singular rotatory deformation at different defined shear rates was applied. Cooling simulates the extrusion process, where the material is also first heated, then cooled in the die section, and subsequently subjected to laminar flow, which exhibits much smaller shear rates than the screw section; apparent shear rates of 1.1–46 s^−1^ were calculated for the die section in various works on HME [59]. In the present work, an apparent shear rate between 8.6 to 10.9 can be calculated according to Son [60], depending on whether the outer rounded part of the flow channel is considered in the calculation or not. It should be mentioned that shear rates are not constant across the cross-section, but have a distribution that depends on the velocity gradients; they can be much smaller or higher than calculated [61].

**Figure 10:**
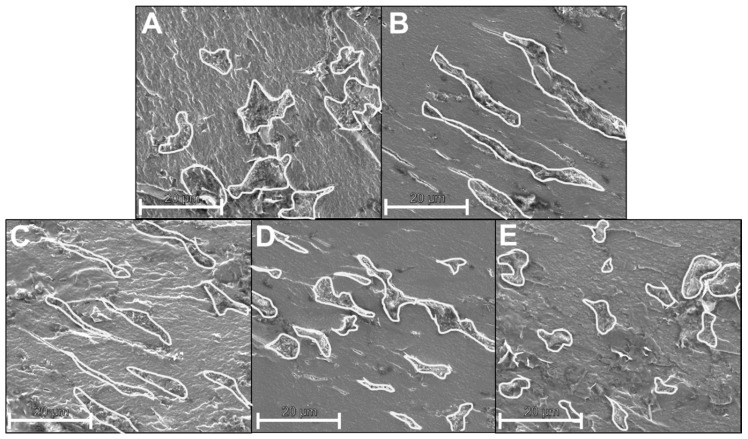
SEM-images of pellets from SPI + 30% WPC, which underwent a defined, extrusion-like thermal treatment at 125 °C, were cooled to 90 °C and then deformed for a given shear rate in the CCR: (**A)** 0 s^−1^, (**B**) 0.25 s^−1^, (**C**) 1 s^−1^, (**D**) 2.5 s^−1^, (**E**) 10 s^−1^.

The influence of the shear rate of a singular deformation on the morphology of the dispersed WPC phase can be seen in Figure 10.

The dispersed phase was outlined with bright lines to improve visibility. At a shear rate of 0 s^−1^, the dispersed phase has a relatively compact, roundish shape. At 0.25 s^−1^, the phase begins to deform and forms an elongated shape oriented in the direction of deformation. As the shear rate continues to increase (1 s^−1^), the dispersed phase retains its elongated, aligned shape. At 2.5 s^−1^, however, shapes develop that are still deformed and aligned, but shorter than at 1 s^−1^. With still further increasing shear rate (10 s^−1^), an elongated shape is no longer evident, but more compact and visibly smaller shapes than in the initial situation at 0 s^−1^.

The results thus indicate that with increasing shear rate, the dispersed WPC phase first adopts a deformed shape, but with further increasing shear rate it is then broken up.

This behaviour has already been demonstrated for different polymer blends, e.g., by Hanafy et al. [62] with a 70:30 blend of polycarbonate and styrene-co-acrylonitrile random copolymer: a deformed shape of the dispersed phase and then a breakup could be observed with increasing shear rate.

For the extrusion process, the results imply that the thermomechanical stress can not only lead to a break-up and thus to a comminution of the dispersed WPC phase, but also to a deformed, elongated shape of the dispersed WPC phase. This deformed shape was also detected, for example, in SPI-pectin blends treated by the shear cell [63] and could be attributed to the low interfacial tension in the system.

Overall, it is assumed that due to the high shear rates and the inhomogeneous flow field in the screw section, the dispersed WPC phase is broken up and arrives as very small particles (<1 µm) in the die section. Due to the laminar flow field in the die section, these small particles could then deform and align themselves in the flow direction, analogous to the shape of the overlying water-rich dispersed phase. However, depending on the shear rate, this could also lead to further breakup.

This insight into the size of the dispersed WPC phase leads to the assumption that the effect of WPC on the over-lying multiphase morphology is rather an indirect effect by being finely dispersed throughout the extrudate, and interacting with the morphology of the multiphase system by influencing the rheological properties and water distribution.

Nevertheless, the morphology of this small dispersed WPC phase could also play a certain role, as it can affect the rheological behaviour of the blend. For instance, Filipe et al. [64] have shown for polymer blends that a system with a deformed, non-round dispersed phase exhibits different rheological properties than the same system with a round dispersed phase.

## 4. Conclusions

The addition of WPC as a model protein in two different concentrations had a significant influence on high moisture extrusion processing and the resulting extrudates. With increasing WPC concentration, the anisotropic structures became more pronounced, the cutting force decreased and the *AI* increased. The achieved *AI* values were comparable to values from the literature, with SPI + 30% WPC yielding even higher *AI* values. The die pressure in the process decreased significantly with increasing WPC content, suggesting that WPC addition led to lower blend viscosity. The multiphase morphology was distinctively affected by the WPC addition. The structures became thinner and were more evenly distributed; the apparent V-shape caused by the laminar flow in the die section was preserved but had a flatter angle. In the SPI matrix, the WPC forms a dispersed phase, the morphology of which depends on the (thermo-)mechanical stresses. Since the WPC dispersed phase is significantly smaller than the over-lying structure of the SPI (water-rich dispersed phase and water-poor continuous phase), it is assumed that the WPC acts on the multiphase morphology via influencing the rheological properties and the water distribution. 

These findings show that the blending of protein-rich raw materials has the potential of tailoring the product structure of extruded meat analogues. The blending of materials from plant and animal origins, besides its advantages on the nutritive value and sensorial properties, can also improve the texture of meat analogues. Raw material selection for blending should be based on how rheological properties and water distribution are affected.

## Figures and Tables

**Table 1:** Temperature profiles of the extrusion trials. Temperature of the barrels 6–8 and the die adapter were specifically adjusted to achieve material temperatures of 115, 125 and 133 °C for each material system.

T_Material_/°C	115			125			133		
mixture	SPI	SPI + 15% WPC	SPI + 30% WPC	SPI	SPI + 15% WPC	SPI + 30% WPC	SPI	SPI + 15% WPC	SPI + 30% WPC
T_Barrel,6–8 and die_/°C	133	138	138	144	149	150	155	159	160
